# Short‐ and long‐term outcomes of IPAA for ulcerative colitis in Latin America: A retrospective, multicentre assessment

**DOI:** 10.1111/codi.70518

**Published:** 2026-06-10

**Authors:** Surgical IBD LATAM Consortium, Pamela Jacinto, Karina Collia Ávila, Mariana Coraglio, Henrique Sarubbi Filmann, Juan Pablo Muñoz, Marcos Riccardini, Rafael García Duperly, Eduardo Londoño‐Schimmer, Andrés Iglesias, Nicolás Rotholtz, Camila Brass Harriott, Gustavo Rossi, Juan Pablo Campana, Juan Ricardo Marquez, Adriana Cherem Alves, Augusto Carrie, Lucio Sarubbi Fillmann, Mariano Cillo, Nicolás Avellaneda, Claudio Saddy Rodrigues Coy, Rogerio S Parra, Omar Féres, Rogerio Saad Hossne, Felipe Bellolio, Antônio Lacerda Filho, Bruno Augusto Alves Martins, Carlos Ramon Silveira Mendes, Eron Fábio Miranda, Paulo Gustavo Kotze

**Keywords:** ileoanal pouches, inflammatory bowel diseases, restorative proctocolectomy, ulcerative colitis

## Abstract

**Introduction:**

Data on ileal pouch‐anal anastomosis (IPAA) outcomes in ulcerative colitis (UC) patients from resource‐constrained settings, such as Latin America, are limited. This study aimed to evaluate demographic characteristics, perioperative outcomes and long‐term results of UC patients undergoing IPAA in Latin America.

**Methods:**

A retrospective analysis was conducted on UC patients who underwent IPAA at 11 academic centres across Argentina, Brazil, Chile and Colombia between 2012 and 2022. Main outcome was 30‐day postoperative complications.

**Results:**

A total of 273 patients underwent IPAA; 49.8% were female, with a mean age of 37.5 years. Three‐stage IPAA was performed in 68.9% of cases, while 31.1% underwent a two‐stage approach. A minimally invasive technique was used in 50% of cases, with a 4% conversion rate. The 30‐day complication rate was 36.6%, and 30‐day mortality was 0.7%. On multivariate analysis, use of advanced therapies within 12 weeks of surgery was protective against postoperative complications (OR 0.24, *p* = 0.008), whereas ASA III/IV status was independently associated with higher complication rates (OR 5.14, *p* = 0.043). Long‐term follow‐up was available for 176 patients (median 60 months). IPAA‐related complications occurred in 31.5%, including pouchitis, strictures, cuffitis and fistula formation. Pouch failure was observed in 8.5%.

**Conclusion:**

IPAA for UC in Latin America shows acceptable morbidity, low mortality and long‐term outcomes comparable to high‐resource settings, supporting its feasibility in resource‐constrained environments.

**What does this paper add to the literature?**

This paper represents one of the largest multicentre series to date evaluating IPAA outcomes in patients with ulcerative colitis from Latin America. By analysing real‐world outcomes in a resource‐constrained setting, this study highlights the feasibility of delivering complex surgical care for IBD patients outside high‐income countries, thereby contributing valuable insights to the global literature.

## INTRODUCTION

Ulcerative colitis (UC) is a chronic inflammatory bowel disease (IBD), and despite advancements in medical therapy, a subset of patients requires surgical intervention due to refractory disease, complications or dysplasia. Restorative proctocolectomy with ileal pouch‐anal anastomosis (IPAA) has become the gold standard procedure for patients undergoing surgery for UC, providing favourable outcomes in terms of bowel function and quality of life [1]. Although extensive data on IPAA outcomes are available from developed countries [2, 3, 4, 5], reports from low‐ and middle‐income countries (LMICs) remain scarce.

Most studies on IPAA outcomes originate from North America, Europe and high‐income regions of Asia, where healthcare systems provide advanced surgical techniques, structured follow‐up care and access to specialised centres. These studies have characterised key demographic and clinical factors influencing outcomes, including postoperative complications, pouch survival and long‐term functional results [6, 7]. However, the experience and outcomes of patients undergoing IPAA in resource‐constrained settings, such as Latin America, remain underexplored, with most available data limited to single‐centre reports involving small patient cohorts [8, 9]. Given the socioeconomic, healthcare and genetic differences in these populations, findings from high‐income regions may not be directly generalisable to all regions of the globe.

Latin America represents a diverse and dynamic region with evolving healthcare systems and increasing rates of IBD, mirroring trends observed in more industrialised nations [10, 11]. Despite these developments, disparities in access to specialised care and surgical expertise persist, potentially impacting outcomes for complex procedures like IPAA. The limited data from LMICs hinder the ability to make evidence‐based recommendations tailored to these populations and highlight the pressing need for region‐specific research. Understanding the demographic characteristics and clinical outcomes of IPAA in Latin America is essential to improving surgical care and patient management.

Our study addresses this knowledge gap by presenting the demographics, perioperative outcomes and long‐term results of UC patients undergoing IPAA in Latin America. By comprehensively analysing this population, we aim to contribute to the global understanding of surgical outcomes in UC while emphasising the unique challenges and opportunities present in LMICs. Our findings seek to inform clinical practice and guide future research in this critical area.

## METHODS

### Design and setting

Consecutive patients with UC who underwent IPAA at 11 IBD‐specialised academic centres across Latin America (Argentina, Brazil, Chile and Colombia) between 2012 and 2022 were included in this study.

**FIGURE 1:**
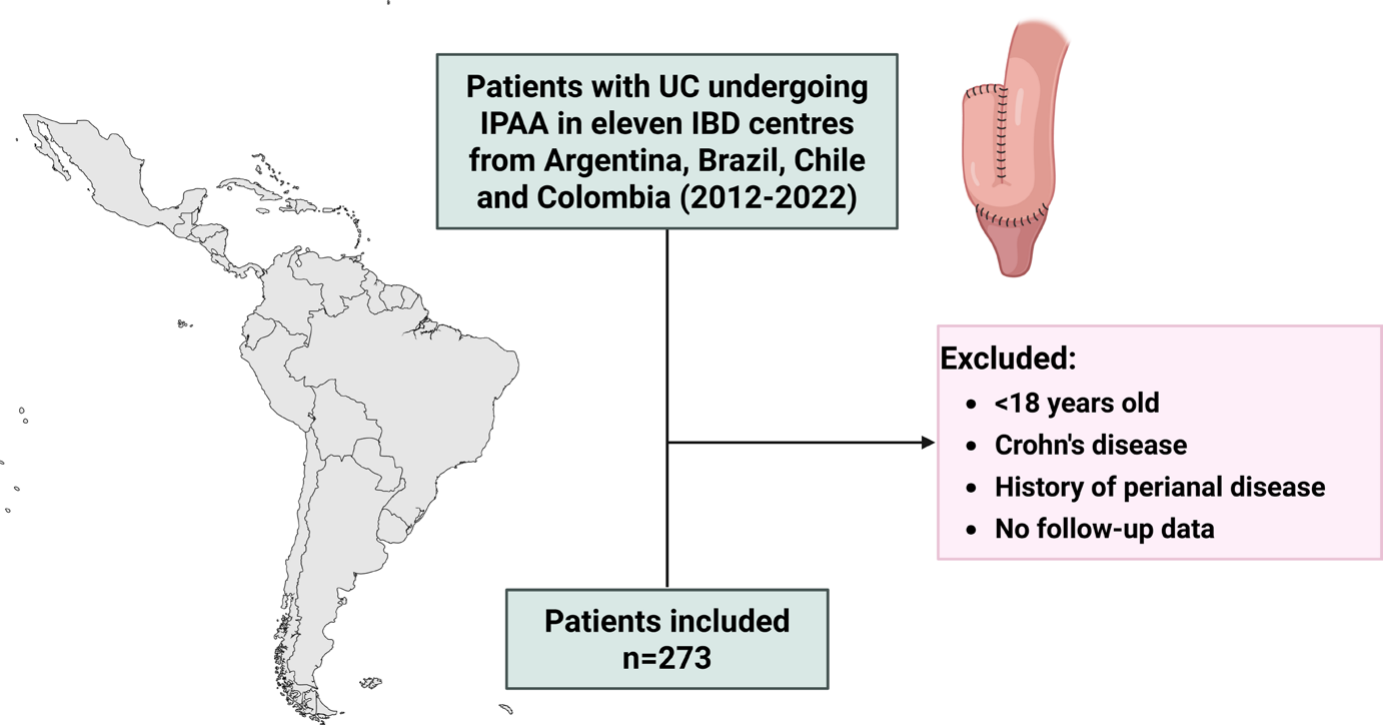
Patient selection flow chart. Created in https://BioRender.com.

Inclusion criteria were patients aged over 18 years with a confirmed histological diagnosis of UC who underwent proctocolectomy with IPAA creation during the study period, performed in one, two or three stages. Indications for surgery included colonic dysplasia and medically refractory disease. Patients operated on for Crohn's disease, those with UC who did not receive an IPAA, and patients with a history of perianal disease were excluded. Figure 1 shows the patient selection flowchart.

A retrospective analysis of medical records was conducted, and the collected data were entered into a database specifically created for this study using REDCap®, in compliance with international standards for the protection of personal information.

### Variables

Data on comorbidities and previous exposure to medical treatment were collected to identify differences between groups before outcome analysis.

- *Preoperative variables* included: age, gender, Charlson comorbidity index, smoking status, body mass index (BMI), presence of preoperative anaemia, serum albumin levels, ASA score and history of previous abdominal surgeries. Disease‐related variables included time from UC diagnosis to surgery, exposure to advanced therapies prior to surgery and exposure within 12 weeks of the operative procedure. Advanced therapies refer to biologics and small molecule oral medications that are typically used in moderate to severe forms of Crohn's disease and UC [12].
- *Intraoperative variables* included: operating time, surgical urgency (elective vs. emergency), operative approach (minimally invasive vs. open), conversion rate, intraoperative complications and anastomotic characteristics.
- *Postoperative factors* included: length of hospital stay, complications stratified using the Clavien–Dindo classification (CDC) [13], readmission and reoperation rates, and 30‐day mortality. Major complications were defined as CDC ≥III [14].
- *Long‐term follow‐up* variables included: time from IPAA creation to last follow‐up visit, presence of pouch‐related complications (i.e., pouchitis, strictures, fistulas), and pouch survival, defined as the patient maintaining a functional pouch at the last follow‐up.

### Outcomes

The main outcome was 30‐day postoperative complications. Secondary outcomes were rates of minimally invasive approach (robotics or multiport laparoscopy), reoperations, rehospitalisations, 30‐day mortality, long‐term complications related to the pouch, and the rate of IPAA failure.

### Statistical analysis

A convenience sample size was used. Categorical variables were summarised as numbers and percentages (%), and the Chi‐squared test or Fisher's test (when appropriate) was used for comparison. Continuous variables are expressed as mean and standard deviation (SD) or median and interquartile range (IQR) according to distribution. If normality was assumed, they were compared using the Student's *t*‐test, and the Mann–Whitney test was used otherwise.

For the risk of surgical complications, univariate and multivariate analyses were performed using logistic regression, with the odds ratio (OR) and its 95% confidence interval (95%CI) calculated. In the multivariate analysis, those variables considered clinically relevant by the authors were included.

To calculate IPAA failure‐free time, the Kaplan–Meier method was used with an estimate of 10‐year survival and its corresponding 95%CI, the time during which at least 15% of the population remains at risk.

A *p*‐value less than 0.05 or an OR whose CI does not contain the value 1 was considered significant. The software used was SPSS 22.0® (IBM Corp., Armonk, NY, USA).

### Ethical considerations

This study was conducted in accordance with the Declaration of Helsinki, the International Conference on Harmonisation Good Clinical Practice (ICH‐GCP) guidelines, and applicable national and institutional research ethics regulations.

Given the non‐interventional, observational design and exclusive use of anonymised data, the requirement for written informed consent was waived in countries where permitted by law. In jurisdictions where informed consent was required, it was obtained in accordance with local institutional review board (IRB) or ethics committee protocols. Ethics approval was obtained at all participating centres prior to patient enrolment.

No experimental or investigational procedures were performed. All surgical and perioperative management decisions were made by the attending clinical teams according to institutional standards of care. The study involved no deviations from routine clinical practice, and patients were not subjected to additional risks due to study participation.

To safeguard confidentiality, only de‐identified data were collected. Each site anonymised data prior to entry into the centralised database. The electronic data capture system (REDCap®) was hosted on a secure server at CEMIC University Hospital, Buenos Aires, Argentina, with access restricted to authorised personnel via encrypted, password‐protected logins. The platform maintains a complete audit trail and is compliant with international data protection standards, including the General Data Protection Regulation (GDPR), where applicable.

Regional investigators coordinated oversight of data integrity and protocol compliance, and data monitoring processes were implemented to ensure accuracy, completeness and consistency across participating sites.

## RESULTS

### Clinical characteristics

A total of 273 patients who underwent IPAA for UC at the participating centres during the study period were included. Of these, 136 (49.8%) were female, and the mean age was 37.5 years (SD 14). Most patients had a low Charlson comorbidity index, and the median BMI was 22.5 kg/m^2^ (SD 5.2), with only 4.9% classified as obese.

**FIGURE 2:**
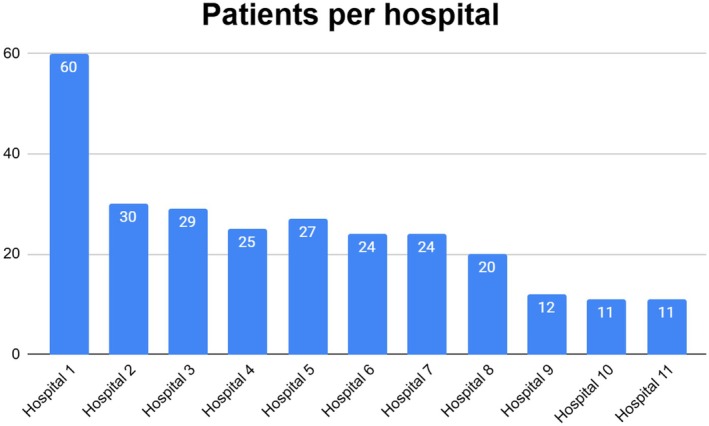
Patients recruited in each hospital participating in the study.

Table 1 summarises preoperative and intraoperative characteristics, and Figure 2 shows the distribution of patients across the participating centres.

**TABLE 1:** Preoperative and intraoperative variables.

	All patients (*n* = 273)
Female, *n* (%)	136 (49.8)
Age (years, mean ‐ SD)	37.5 (14)
Smoking, *n* (%)	24 (8.8)
Charlson comorbidity score, *n* (%)	
0–1	962 (96.3)
2–3	31 (3.1)
>3	6 (0.6)
BMI (mean, SD)	22.5 (5.2)
Obese, *n* (%)	8 (4.9)
Missing, *n* (%)	111 (40.7)
Preoperative Anaemia (%)	123 (45.1)
Preoperative albumin (mean, SD)	3.9 (0.72)
Low albumin (<3 g/dL), *n* (%)	22 (8.1)
Missing, *n* (%)	90 (33)
Previous abdominal procedures, *n* (%)	164 (60.3)
Missing, *n* (%)	1 (0.37)
Time from diagnosis to surgery (years, mean, SD)	8 (8.4)
Missing, *n* (%)	4 (1.5)
Previous exposure to advanced therapies, *n (%)*	126 (46.2)
Time from starting advanced therapies to surgery (months, median, IQR)	18 (8–36)
Missing, *n* (%)	2 (0.73)
Number of advanced therapies received prior to surgery, *n* (%)	
1	72 (57.1)
2	36 (28.6)
>3	18 (14.3)
ASA score, *n* (%)	
I	82 (30)
II	164 (60.1)
III	15 (5.5)
Operating time (minutes, mean, SD)	226 (70)
Missing, *n* (%)	36 (13.2)
Minimally invasive approach, *n* (%)	141 (51.6)
Conversion rate	11 (4)
Intraoperative complications, *n* (%)	18 (6.6)
IPAA stages, *n* (%)	
2‐stage	85 (31.1)
3‐stage	188 (68.9)
Type of anastomosis, *n* (%)	
Hand‐Sewn	15 (5.5)
Stappled	258 (94.5)

Abbreviations: ASA, American Society of Anaesthesiologists physical status; BMI, body mass index; IPAA, ileoanal pouch anastomosis.

Regarding disease‐related variables, the mean time from diagnosis to surgery was 8 years (SD 8.4). Nearly half of the patients had been exposed to at least one line of advanced therapy before surgery, with a median time from initiation of therapy to surgery of 18 months. None of the patients was using corticosteroids at the time of IPAA construction. Overall, 68.9% underwent a three‐stage IPAA, and 31.1% a two‐stage procedure.

### Operative details

At the time of surgery, 90.1% of patients were classified as ASA I or II. The mean operating time was 226 min (SD 70). A minimally invasive approach was used in 51.6% of cases, with a conversion rate to open surgery of 4%. Intraoperative complications occurred in 18 patients (6.6%), including bleeding and small bowel injury. Most IPAA constructions (94.5%) were stapled, with 5.5% hand‐sewn.

### Primary outcomes

Overall, 100 patients (36.6%) experienced complications within 30 days postoperatively. Of these, 59% were classified as minor and 41% as major complications. Regarding the most common complications, 28 patients presented postoperative pelvic abscess, 20 patients suffered a prolonged postoperative ileus, 13 patients had abdominal obstruction after surgery, nine patients had fever, six patients had bleeding and the rest of the patients had other complications, including urinary tract infection, pneumonia, ileostomy complications and ureteric injuries.

### Postoperative outcomes

Table 2 summarises the main postoperative outcomes. The median hospital stay was 6 days (IQR 5–9), and 5.1% of patients required prolonged ICU admission. The reoperation and readmission rates were both 9.5%. The most common indications for reoperation were intestinal obstruction (28%), anastomotic leak (25%), bleeding (9.4%), fascial dehiscence (9%), abdominal abscess (8%) and other causes (20.6%).

**TABLE 2:** Short‐term postoperative outcomes.

	All patients (*n* = 273)
Hospitalisation days (median, IQR)	6 (5–9)
Missing, *n* (%)	33 (12.1)
Prolonged requirement of ICU, *n* (%)	14 (5.1)
Complications, *n* (%)	100 (36.6)
Minor	59 (59)
Major	41 (41)
Reoperation, *n* (%)	32 (11.7)
Readmission, *n* (%)	26 (9.5)
Mortality, *n* (%)	2 (0.7)

Abbreviations: ICU, intensive care unit.

Two patients (0.7%) died within 30 days of the procedure.

### Long‐term outcomes

Following IPAA, 134 patients (49.1%) underwent pouchoscopy, with a mean interval of 7.4 months (SD 3.2) from surgery. Endoscopic findings were normal in 85 patients (63.9%); pouchitis was identified in 36 patients (27.1%), and mucosal erosions or ulcers in 12 patients (9%).

Long‐term follow‐up was available for 176 patients, with a median follow‐up duration of 60 months (IQR 26–99). During this period, 73 patients (31.5%) developed IPAA‐related complications, with a mean time to onset of 18 months (SD 25). The most frequent complications were pouchitis (42.5%), anastomotic strictures (18%), cuffitis (12%) and pouch‐related fistula (7%).

**FIGURE 3:**
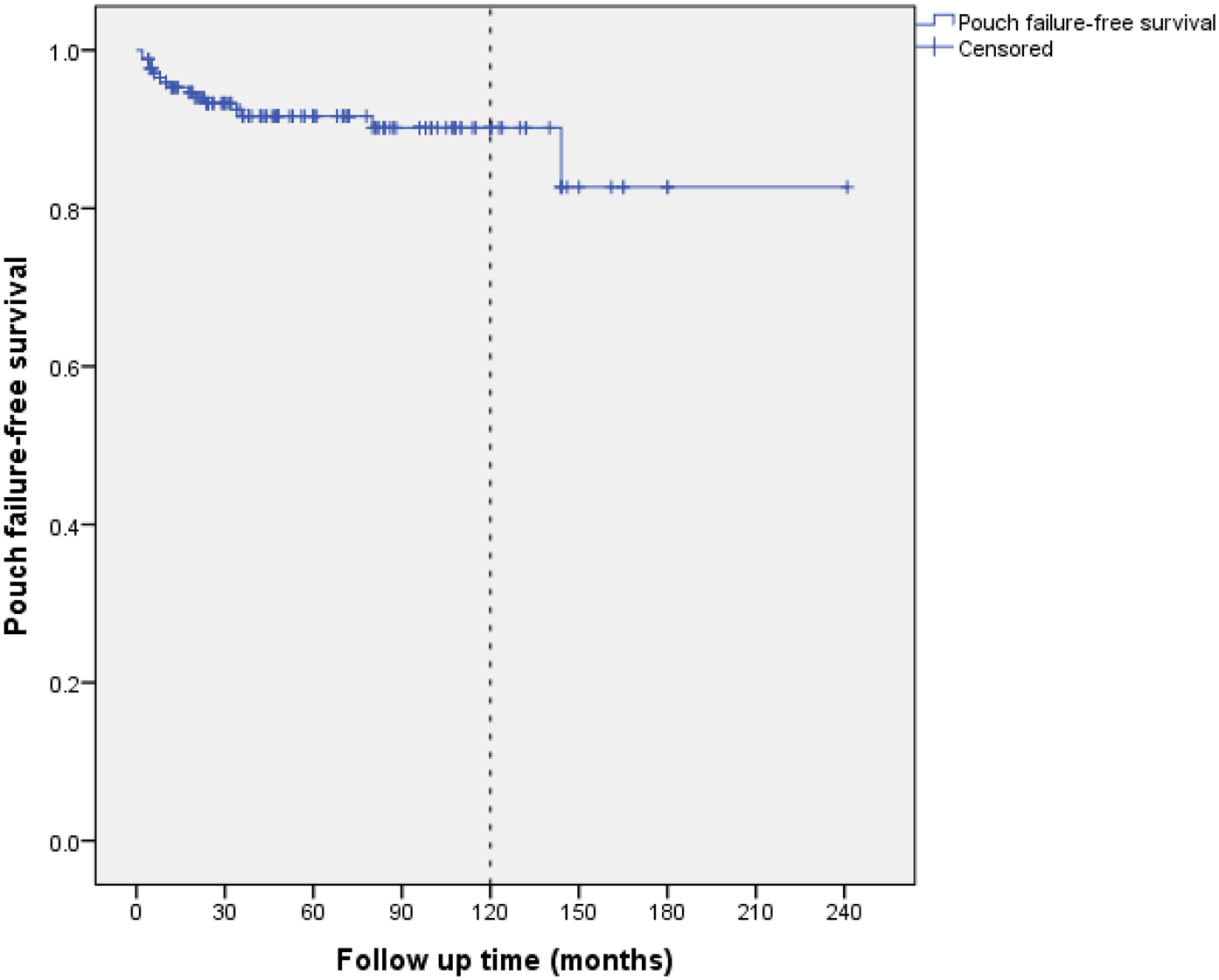
Estimated IPAA survival over time.

Figure 3 illustrates the IPAA survival curve for the cohort. Pouch failure occurred in 15 patients (8.5%) at a mean of 27 months (SD 38) postoperatively. Treatments for pouch failure included diverting ileostomy in nine patients and pouch excision with end ileostomy in six patients; no redo pouches were performed. The estimated 10‐year pouch survival rate was 90.2% (95% CI, 85.1–95.3).

### Multivariate analysis

In multivariate analysis, advanced therapy use within 12 weeks of surgery was associated with a reduced risk of postoperative complications (OR 0.24, *p* = 0.008), while ASA III/IV status was independently associated with an increased risk of complications (OR 5.14, *p* = 0.043). Full results are presented in Table 3.

**TABLE 3:** Multivariate analysis considering postoperative complications as the dependent variable.

Variables	OR	Standard error	*p*	95%CI
Age	0.99	0.12	0.592	0.96–1.02
Female sex	2.04	0.99	0.141	0.79–5.27
Smoking	2.56	2.02	0.233	0.55–12
Preoperative anaemia	1.32	0.62	0.548	0.53–3.30
Previous abdominal procedure	2.44	1.30	0.095	0.86–6.96
Perioperative biologics	**0.24**	**0.13**	**0.008**	**0.08–0.70**
Conversion to open surgery	0.43	0.44	0.407	0.06–3.16
Intraoperative complications	1.30	1.12	0.760	0.24–7.08
3‐stage IPAA (vs. 2‐stage)	0.85	0.41	0.737	0.33–2.20
High BMI	1.06	1.08	0.950	0.15–7.71
ASA III/IV	**5.14**	**4.16**	**0.043**	**1.05–25.10**

*Note*: Bold‐faced values indicate statistically significant at alpha <0.05.

Abbreviations: ASA, American Society of Anaesthesiologists physical status; BMI, body mass index; IPAA, ileoanal pouch anastomosis.

## DISCUSSION

This multicentre retrospective study provides a comprehensive analysis of the short‐ and long‐term outcomes of IPAA for UC in Latin America, contributing to the limited body of evidence on this topic from LMICs. The findings highlight both the feasibility and challenges of performing IPAA in a region with diverse healthcare systems and evolving IBD management practices.

Considering the various challenges of implementing minimally invasive surgical programmes in LMICs, it is commendable that approximately half of the patients in this cohort underwent a laparoscopic approach. A retrospective study of patients undergoing IPAA for UC between 2011 and 2022 at the University Hospitals of Leuven revealed that 86% of these cases were performed laparoscopically [15]. This finding suggests that the initial Latin American experience may develop and converge with the outcomes reported in other high‐volume referral centres. Although there is a trend toward a significant increase in robotic colorectal procedures throughout LATAM [16], only three robotic IPAAs were noted in this cohort. This may suggest that the adoption of robotic surgery is experiencing a delay comparable to the one seen when the laparoscopic approach was first introduced in IBD surgery [17].

Almost 70% of the patients in this cohort had a three‐stage IPAA. A staged restorative proctocolectomy seeks to reduce the devastating complications of an anastomotic leak. It also allows confirming UC in the surgical specimen, nutritional optimisation and tapering of steroids [18]. Although recent evidence suggests that a modified two‐stage IPAA may be associated with fewer complications and a shorter length of stay compared to three‐stage or standard two‐stage IPAA [1], Latin American surgeons have not yet adopted this approach. One possible reason is the need for diligent postoperative follow‐up in modified two‐stage IPAA cases to detect potential complications early, which can be difficult to achieve in some healthcare settings.

The overall postoperative complication rate of 36.6% observed in this study is consistent with previously reported rates from high‐income regions [19, 20]. Baek and colleagues evaluated short‐term outcomes in 588 patients who underwent laparoscopic IPAA at the Mayo Clinic in Rochester, Minnesota, between April 1999 and July 2012. 93.9% of procedures were performed for UC, and complications occurred in 36.9% of patients; no mortality was reported [19]. In Australia, Lim and colleagues reported a 34.9% rate of early complications among 212 consecutive UC patients who underwent IPAA at the Royal Brisbane and Women's Hospital between 1990 and August 2016 [20].

Although the postoperative morbidity rate was comparable with previously reported rates from high‐income areas, the proportion of major complications (41%) was relatively high. In the previously presented Mayo Clinic and Royal Brisbane cohorts, only 9.7% of patients had major complications [19, 20]. This suggests that the burden of severe morbidity may be more pronounced in LMIC settings. This may be partially explained by the high proportion of patients undergoing surgery for advanced disease (delayed surgical indication) and by prior exposure to multiple lines of medical therapy, a phenomenon described in other LMIC IBD cohorts [21].

Interestingly, using advanced therapies within 12 weeks before surgery was identified as a protective factor against early postoperative complications in the multivariate analysis (OR: 0.24, *p* = 0.008). This controversial matter has been explored before, with some studies suggesting that the use of perioperative advanced therapies is not associated with more complications [22]. In contrast, other studies have found a positive association between these agents and postoperative adverse events in IBD patients [23, 24]. The analysis of perioperative advanced therapies was limited by the lack of detailed data on specific agents across centres, precluding subgroup analyses by biologic class. Therefore, the observed protective association should be interpreted with caution and may reflect broader aspects of perioperative optimisation rather than a direct pharmacological effect. Further prospective studies are needed to confirm this association and clarify the role of biologics in perioperative outcomes in resource‐constrained environments.

The current report on the rate of pouch survival (estimated to be 90.2% at 10 years) is comparable to previous reports from other regions [25, 26]. Notably, no redo pouches were documented in this cohort, which may reflect limited access to tertiary centres with specialised surgical expertise in the region. A Swedish population‐based cohort study of 1796 UC patients who underwent IPAA reported 10‐year pouch survival rates of 94% for primary IPAA and 92% for secondary (subsequent to a previous ileorectal anastomosis). Two patients underwent a redo pouch [25]. Heuthorst and colleagues conducted a systematic review and meta‐analysis that included 30 studies, comprising a total of 22,978 patients who underwent IPAA, of whom 20.839 had UC. They observed pooled pouch failure rates of 7.8% and 10.3% after a median follow‐up of ≥5 and ≥10 years following IPAA, respectively [26].

Regarding long‐term complications, pouchitis emerged as the most frequent adverse event (42.5%), which is consistent with global literature [27]. However, the relatively low rate of endoscopic pouch assessment (49.1%) suggests that subclinical inflammation may have been underdiagnosed in this population. Implementing structured surveillance protocols and improving access to pouchoscopy could enhance early detection and management of pouch‐related complications.

This study has several limitations that must be acknowledged. First, it is a retrospective analysis, which is inherently subject to selection bias, missing data and the limitations associated with retrospective data collection. Although the study included patients from 11 specialised centres across four countries, nearly 20% of the cohort came from a single centre, and the participating institutions are predominantly academic and high‐volume centres. Consequently, the findings may not be generalisable to all healthcare settings in Latin America, particularly to lower‐volume or non‐academic hospitals. As referral IBD‐specialised academic centres, these institutions are more likely to manage a higher proportion of complex cases requiring IPAA. Accordingly, although full population‐level representation cannot be ensured, this cohort likely provides a robust and pragmatic reflection of real‐world outcomes.

Second, there was heterogeneity among centres regarding surgical techniques, perioperative management protocols, and access to resources such as advanced surgical technologies and postoperative care, which may have influenced outcomes. Standardisation of surgical indications, staging strategies and postoperative follow‐up protocols across centres was not possible, potentially introducing variability in the results.

Third, although long‐term follow‐up data were available for a considerable proportion of the cohort, not all patients completed extended follow‐up, and the potential for loss to follow‐up may have impacted the assessment of long‐term outcomes such as pouch function and survival. Fourth, the relatively low rate of endoscopic pouch surveillance limits the interpretation of subclinical pouch complications, and underdiagnosis of conditions such as asymptomatic pouchitis cannot be ruled out. The absence of a control group from high‐income countries precludes direct comparative analysis and limits the ability to attribute differences specific to the resource‐constrained context. Lastly, some information related to causes associated with reoperations and prolonged ICU was not specified in the database.

Nevertheless, this study has several notable strengths. It represents one of the largest multicentre series to date evaluating IPAA outcomes in UC patients from Latin America, a region where data remain scarce. The inclusion of 11 academic centres across four countries enhances the generalisability of the findings to a broader Latin American context. Additionally, the study provides both short‐ and long‐term outcomes, offering valuable insights into postoperative complications, pouch survival and functional results over an extended follow‐up period. The use of standardised data collection methods and compliance with international data protection standards further strengthen the reliability and validity of the reported results. Finally, by analysing real‐world outcomes in a resource‐constrained setting, this study highlights the feasibility of delivering complex surgical care for IBD patients outside high‐income countries, contributing important knowledge to the global literature.

In conclusion, in this multicentre Latin American cohort, IPAA for UC demonstrated acceptable short‐term morbidity and low mortality, with long‐term outcomes comparable to those reported in high‐income countries. Despite the challenges inherent to resource‐constrained settings, IPAA remains a feasible and effective surgical option. However, the relatively high rate of major complications underscores the need for continued efforts to optimise perioperative care and improve access to specialised surgical expertise. These findings contribute to the growing body of evidence supporting the safe implementation of complex IBD surgery in LMICs and highlight the importance of regional collaborative research to further refine surgical strategies and patient outcomes.

Future efforts should focus on creating regional registries and conducting prospective collaborative studies to further characterise the impact of socioeconomic factors, surgical techniques and perioperative optimisation strategies on IPAA outcomes in Latin America. Additionally, fostering international partnerships and training programs could help bridge the gap in access to advanced surgical care for IBD patients in LMICs.

## FUNDING INFORMATION

No financial compensation was provided for participation in this study, either to the patients or the research team.

## CONFLICT OF INTEREST STATEMENT

No conflict of interests declared by any of the authors.

## ETHICS STATEMENT

Given the non‐interventional, observational design and exclusive use of anonymised data, the requirement for written informed consent was waived in countries where permitted by law. In jurisdictions where informed consent was required, it was obtained in accordance with local institutional review board (IRB) or ethics committee protocols. Ethics approval was obtained at all participating centres prior to patient enrolment. No experimental or investigational procedures were performed.

## AI STATEMENT

No generative AI tools were used in the writing or preparation of this manuscript.

## Data Availability

The data that support the findings of this study are available from the corresponding author upon reasonable request.
